# L'ostéo-chondrome de la région inter-trochantérienne: une localisation rare

**DOI:** 10.11604/pamj.2015.21.149.6683

**Published:** 2015-06-24

**Authors:** Youness Sasbou, Mohammed Boussaidane

**Affiliations:** 1 Université Mohammed V, Faculté de Médecine et de Pharmacie, Service de Chirurgie Orthopédique II, Hôpital Militaire d'Instruction Mohammed V, 10000 Rabat, Maroc

**Keywords:** Ostéo-chondrome, inter-trochantérienne, fémur, Osteo-chondroma, intertrochanteric region, femur

## Image en medicine

**Figure 1:**
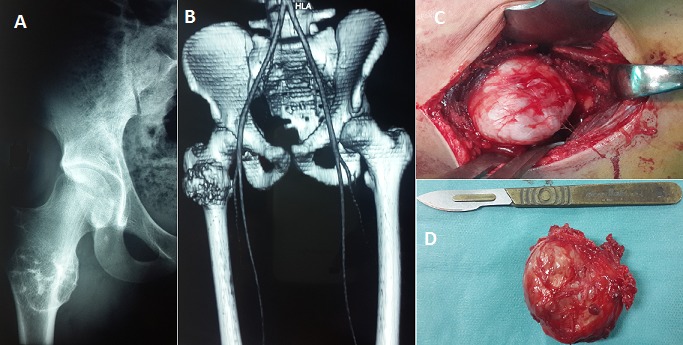
(A) radiographie standard montrant la tumeur; (B) image TDM montrant la localisation inter-trochantérienne; (C) vue per-opératoire montrant la tumeur; (D) photo de la pièce opératoire

L'ostéochondrome ou exostose est la tumeur osseuse primaire bénigne la plus fréquente, elle contient à la fois de l'os et du cartilage et siège le plus souvent au niveau des métaphyses des os longs. Ces tumeurs sont le plus souvent asymptomatiques, en fonction de leur taille, leur emplacement et de leur localisation; leur siège au niveau du fémur proximal est rarement rencontré car la plupart sont généralement asymptomatiques. Nous rapportons un cas rare d'ostéochondrome de la région inter-trochantérienne du fémur chez un patient âgé de 35 ans, présentant depuis 1 année une tuméfaction de la face externe du tiers supérieur de la cuisse droite, indolore, sans notion de traumatisme ni d'antécédents médicaux ou chirurgicaux. L'examen physique a mis en évidence une masse ferme, indolore, immobile, sans signes cutanées associées; la mobilité de la hanche est normale et le reste de l'examen physique, notamment l'examen vasculo-nerveux du membre inférieur droit, est sans particularité. Les radiographies standards (A) et la TDM (B) ont été réalisés pour évaluer le siège, l’étendue et la nature de la tumeur. Le patient a bénéficiée d'une exérèse chirurgicale de la masse (C,D) et l'examen anatomopathologique a confirmé la nature histologique de la tumeur. Le patient a été maintenu en décharge pendant 2 semaines avec reprise de la marche à la 4^ème^ semaine postopératoire. A 12 mois de recul, l'examen clinique et radiographique n'ont pas objectivé de signes de récidive. Figure 1

